# Building resilient Arctic science amid the COVID-19 pandemic

**DOI:** 10.1038/s41467-020-19923-2

**Published:** 2020-12-08

**Authors:** Andrey N. Petrov, Larry D. Hinzman, Lars Kullerud, Tatiana S. Degai, Liisa Holmberg, Allen Pope, Alona Yefimenko

**Affiliations:** 1 International Arctic Social Sciences Association (IASSA), 1227W 27th Street, Cedar Falls, IA 50614 USA; 2 International Arctic Science Committee (IASC), Borgir, Norðurslóð 600, Akureyri, Iceland; 3 University of the Arctic (UArctic), UArctic President Office, GRID-Arendal, 4802 Arendal, Norway; 4 Council of Itelmens of Kamchatka “Tkhsanom”. ARCTICenter 348 ITTC University of Northern Iowa, Cedar Falls, IA 50614 USA; 5 International Sámi Film Institute, 9521 Guovdageaidnu, Norway; 6 Arctic Council Indigenous Peoples’ Secretariat, Fram Centre, 9296 Tromsø, Norway

**Keywords:** Climate sciences, Environmental sciences, Environmental social sciences, Geography, Scientific community

## Abstract

Arctic research faces unprecedented disruptions due to COVID-19. This ‘pause’ gives an opportunity to reflect on the current state and the future of Arctic science and move towards a more resilient, thus equitable, coordinated, safe and locally-embedded Arctic research enterprise. Arctic science has been greatly affected by COVID-19. This comment looks forward to how Arctic science could be conducted in the future.

The Arctic has been at the centre of recent climate-driven changes influencing global climate dynamics, regional weather, and international commerce^1^. Now, the scientific community finds Arctic research capabilities severely limited by travel bans and our own trepidation of becoming vectors transmitting COVID-19. Arctic communities have justifiably asked that travel to their areas be curtailed. The consequences of the prolonged gap in field research will resonate for decades across scientific disciplines, through policy decisions, and into economic investments.

COVID-19 is not just an immediate danger for the Arctic. It will have lasting effects on communities as the current health, food security, and economic issues become exacerbated. Remote Arctic villages are poised to experience significant economic losses (including the earnings from hosting science operations), endure reduction of transportation accessibility, and may also face the loss of key knowledge holders—including elders—and thus the loss of culture, heritage, and tradition.

This article, co-written by Indigenous natural and social science experts, represents a synthesis of perspectives from the International Arctic Science Committee (IASC), the International Arctic Social Sciences Association (IASSA), and the University of the Arctic (UArctic) to help guide the science community’s response to the COVID-19 pandemic in the Arctic. We argue that although Arctic research has been disrupted, this pause is giving us a unique opportunity to reflect on the current state and the future of Arctic science and work on building a more resilient, equitable, coordinated, safe, and locally embedded Arctic research enterprise.

## COVID-19 impacts on Arctic science

### Arctic context for COVID-19

The Arctic is home to more than four million people, including Indigenous Peoples. Most remote settlements in the Arctic possess limited, if any, health-care facilities. They also have constrained financial and public resources and often suffer from food security issues and overcrowded housing^2^. Many of the smaller villages are not equipped with civic infrastructure, making it difficult to implement COVID-19 preventive measures. Connectivity of rural communities by transport and by Internet is often poor and limits opportunities for medical evacuation or telemedicine. Consequently, Arctic regions are particularly vulnerable to COVID-19.

Pandemics in the Arctic were disastrous in the past, so communities are now rightfully wary of any outside visitors. Multiple reports indicate that during COVID-19 the Indigenous Peoples have used traditional knowledge and lessons learnt from past pandemics. For example, Dene and Inuit communities in the Canadian Arctic encouraged their members to go on the land to ensure food security and maintain their health^3^. During COVID-19 isolation, a UArctic network of Indigenous colleges from Kola, Taimyr, Yamal, Nenets, Komi, and Sakha used their partnership to create online learning solutions that share knowledge on reindeer husbandry, Indigenous business, fishing, and tourism. A new Arctic Indigenous Virtual Arts Network (AIVAN) was established to connect Indigenous artists. The International Sámi Film Institute developed 15 short films to document the impact of COVID-19 on Indigenous Peoples and Indigenous knowledge-based responses in northern Sweden, Finland, Norway, and Russia. The films will be part of the Arctic Indigenous Film Academy and Film Witness. Building on Western science and Indigenous knowledge together to address the COVID-19 pandemic will be important in the coming months and years.

### Impacts on field research and observations

Even in the best of times, field research in the Arctic regions is quite limited, with sparse observations of atmospheric, marine, and terrestrial variables, biological processes, and social systems^4^. Some research that is able to utilize satellite data may well be able to continue largely uninterrupted, but vital in situ field research has been severely impaired. The consequences of a lost season of field observations will propagate through all Arctic sciences and reverberate globally.

Although research in natural sciences is often conducted in remote regions away from settlements, almost all of these field studies have been cancelled or majorly modified over the anxiety of carrying the virus to regional hubs by science expeditions coming from outside the region. Ocean cruises that wouldn’t have visited any communities have also been cancelled to protect members of the scientific parties. Simultaneously, research by local community experts has also been restricted by quarantine measures. Although some found creative solutions, like in Svalbard, where the out-of-job tourist guides were remotely provided with basic training to do the annual reindeer monitoring, only limited information was collected. There are already successful examples of adapting field work to the new reality. The internationally collaborative MOSAiC Expedition, the largest Arctic research expedition ever, solved the challenges by modifying the logistics and part of its research programme^5^. Yet, further action needs to be taken to develop more resilient research in the Arctic and to avoid loss of data and knowledge, while being responsive to the needs and security of local communities.

## An opportunity for reflection

Extreme events like the COVID-19 pandemic require immediate actions, but may also give time for reflection. The ‘pause’ in the pace of research serves a unique opportunity to scientists to publish and organize existing data, but, most importantly, to take a step back and critically assess the ways in which Arctic science should be conducted. It also may provide a chance for Arctic communities to reflect on the nature of this collaboration given their own priorities and consider what sort of science they will welcome in the future.

We need resilient, adaptive Arctic science that is community relevant and locally embedded, and yet strives to bridge major knowledge gaps of global relevance. Only then will science persist in the Arctic regardless of all disturbances. Strong collaboration, deep trust, and a high level of competence held locally in the Arctic are the sources of resilience that will enable observations, research, and knowledge co-production under dire circumstances, such as this pandemic.

## Recommendations: building resilient Arctic science

### Doing no harm

First and foremost, Arctic scientists must do no harm. Therefore, avoiding travel to Arctic communities to prevent the spread of COVID-19 until all risks are eliminated is essential. In this period, the scientific discovery in the Arctic shall continue, but it will look differently in both the short and long term.

### Embracing the ‘local turn’ and knowledge co-production

**Fig. 1:**
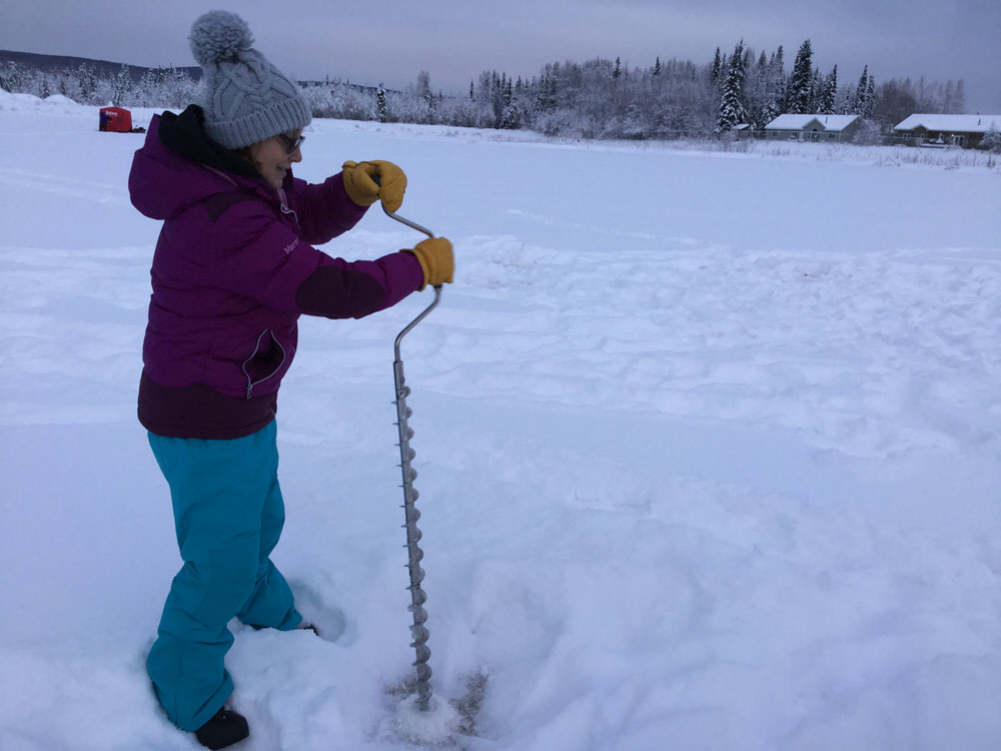
The local turn. Dawn Pomrening, Sidney Huntington School, Galena, Alaska, measures ice thickness with the Fresh Eyes on Ice project (photo by Christopher Arp). This is an example of engaging local observers in collecting data that are of value to a broad array of scientific and forecasting applications while also integrating STEM education into the research process.

The COVID-19 pandemic has re-emphasized that science needs permanent presence in the Arctic. Field stations, community observation systems, local colleges, and community experts reduce the need for outside specialists to conduct observations and maintain instruments (Fig. 1). Thus, investing in Arctic infrastructure is an acute science priority. Re-focusing Arctic science to localized operations is not a simple or inexpensive option, but now is the time to support a broader, localized science infrastructure network that is currently lagging. This investment though needs to be done hand-in-hand with the process of decolonizing Arctic science and embracing Indigenous, traditional, and local knowledge systems.

The need for this ‘local turn’ is very real and long overdue^6^. In Alaska’s Bering Strait region, for example, an Indigenous consortium delivered a scathing assessment of research practices in their homelands^7,8^. The community pointed out that the research has been often initiated, developed, and conducted without consulting the region’s Indigenous Peoples or ensuring their meaningful participation. Scientists sometimes have carried out their projects by disrupting traditional practices, disregarding customs, or overwhelming communities with redundant research that generated minimal feedback. The consequence has been not only a “reproduction of long-standing colonial dynamics”, but poor, inadequate science since “inequitable research can lead to misinformed results, incomplete data, and research fatigue on the part of region residents and communities”^7^.

Although field seasons and travel to the Arctic will continue after the pandemic ends, to build a resilient knowledge-building system in the Arctic, science must rely on local partnerships that are based on equity, trust, and respect. This necessitates collaborating with Indigenous and local communities to co-produce knowledge. Under the leadership of Indigenous Peoples, co-production will lead to improved science because it will be enriched by diverse Indigenous knowledge systems and will give voice to youth and elders. Co-production will redesign, decolonize, and indigenize science–community relationships and develop better, more equitable engagement mechanisms, and, thus, make research a source of empowerment^9^. We are encouraged by a number of successful co-production initiatives, such as the EALÁT Institute^10^ and Indigenous Food Knowledges Network^11^, and hope more will emerge.

As a first step, scientists must ask communities about their concerns and priorities prior to creating research agendas. This means involving Arctic residents and experts remotely to develop research plans and conduct research, providing equipment and fair pay. This requires investing in local capacity-building, along with funding community members to conduct collaborative and independent research, and supporting citizen science, including teachers and students in schools, a contribution that will have a lasting impact. Good examples, such as ELOKA^12^, SmartICE^13^ and ASAD^14^ projects, could serve as blueprints for future locally embedded research initiatives. UIC Science, a science logistics service in Utqiagvik, Alaska, demonstrates how investment in local capacities can provide continuous support for science operations. Scientists should also work with policymakers to strengthen physical and virtual connectivity in the Arctic, while promoting Indigenous Peoples’ control over its use in their communities. Finally, an immediate action must be to ensure that science projects provide emergency relief and long-term support for local contractors, logistics operators, and project participants.

### Focusing on the next generation

A prolonged gap in training will undermine the future of Arctic science. But this opportunity allows us to reflect on how scientists of the future might be better trained. The next generation of scientists, whether from southern-based institutions or residents of the North, must be given the opportunity for place-based learning and training. This applies to traditional academic subjects as well as skills essential to the Arctic. It is important to develop programmes, courses, projects, and research opportunities that focus on Indigenous and local communities’ contexts and encourage learning about the social, historical, political, and economic circumstances in the Arctic.

### The Arctic to remain an essential arena for global research

The economic and social ramifications of the COVID-19 pandemic are likely to persist for years to come. However, rapidly changing environmental impacts make in situ scientific studies vital to continue furthering our knowledge. Personal experiences in the field, community-driven knowledge co-production, and hands-on learning and teaching remain essential to secure insight for coming generations of Arctic experts, whether local or from outside the region. It is critical that the collective research community continues to build understanding of the Arctic as a grand challenge important to humankind.

Resilient Arctic science in the post-COVID-19 world will be based on a global coalition and meaningful collaboration among scientists, local and Indigenous rights-, stake- and knowledge holders, policymakers, science advocates, citizen scientists, industry partners, research institutions, and funding agencies, among others. Further embracing international cooperation^15^, fostering community-science and public–private partnerships, and pursuing well-coordinated planning will pave the way to novel, bold global initiatives for Arctic science, such as a new International Polar Year-2033, which, however, should be based on the locally focused principles and priorities.
